# Biomedical Hydrogels Based on Natural Polysaccharides: Structural Design

**DOI:** 10.3390/gels12070578

**Published:** 2026-06-29

**Authors:** Zezheng Liu, Xin Huang, Jinjin Tong, Hua Zhang

**Affiliations:** 1 College of Veterinary Medicine, Beijing University of Agriculture, Beijing 102206, China; liuzezheng@bua.edu.cn; 2 College of Animal Science and Technology, Beijing University of Agriculture, Beijing 102206, China; huangxin@bua.edu.cn (X.H.); tongjinjin@bua.edu.cn (J.T.)

**Keywords:** biomedical hydrogel, natural polysaccharide, cross-linking method, tissue engineering and regenerative medicine

## Abstract

Hydrogels have gained prominence as a class of biomaterials in biomedicine due to their excellent biocompatibility, biodegradability, and high water retention. Among them, hydrogels derived from natural polysaccharides sourced from plants, animals, and microbes are attracting growing interest due to their renewable nature, low toxicity, low immunogenicity, and diverse functional properties. While several recent reviews have addressed polysaccharide-based hydrogels, they have largely focused on isolated aspects—such as 3D bioprinting formulations, double-network mechanical reinforcement, rheological behavior, or single-source polysaccharides—without establishing an integrated framework that links raw material selection, structural diversity, chemical modification, and crosslinking design to clinical translation. This review distinguishes itself by providing a systematic, end-to-end perspective that spans from the structural diversity of plant- and microbe-derived polysaccharides through recent advances in chemical modification and novel cross-linking strategies, to the fine-tuning of physicochemical properties for enhanced therapeutic outcomes. This article provides an overview of the progress made in the emerging biomedical applications and material design of natural polysaccharide hydrogels in terms of raw material selection, chemical modification, cross-linking mechanisms, and functional utilization. It aims to fully explore the potential of these materials and promote integration into advanced biomedical practices.

## 1. Introduction

Hydrogels have emerged as transformative materials in biomedicine and advanced engineering owing to their exceptional water retention capacity, tunable mechanical properties, and stimuli-responsive behavior [1]. As cross-linked polymeric networks with an interconnected porous architecture, they provide highly adaptable platforms for diverse applications, including tissue engineering, drug delivery, and environmental remediation [2,3]. Recent research has increasingly emphasized the design of smart hydrogels with programmable and stimulus-sensitive functionalities [4,5]. Nonetheless, the clinical application of synthetic hydrogels faces significant challenges, such as harsh crosslinking conditions, poor biodegradability, and potential cytotoxic effects [6,7]. These challenges have shifted attention towards natural polysaccharide-based hydrogels as a more biocompatible and sustainable alternative.

Polysaccharide hydrogels, derived from renewable and abundant sources such as chitosan, cellulose, and hyaluronic acid, inherently possess excellent biocompatibility, biodegradability, and bioactivity [8,9]. In contrast to synthetic hydrogels, they closely resemble the extracellular matrix (ECM) and offer natural functionalities, including antimicrobial activity, cell-adhesive domains, and pH-responsive swelling behavior [10,11,12]. These attributes have enabled their broad application in regenerative medicine, controlled drug delivery, and environmental remediation, supporting tissue repair, localized therapeutic release, and sustainable waste management [13]. Collectively, polysaccharide hydrogels constitute a versatile class of materials with broad development potential across multiple sectors.

Despite their versatility, the practical application of polysaccharide hydrogels continues to encounter significant challenges. Key limitations include inadequate mechanical strength under physiological conditions, unpredictable degradation behavior, and poor structural reproducibility during fabrication processes [14,15]. These drawbacks compromise their long-term stability and functional reliability, particularly in biomedical settings. To overcome these obstacles, recent research has focused on improving structural engineering strategies [16,17]. Chemical modifications, such as backbone grafting and the incorporation of charged functional groups, have been employed to enhance both mechanical performance and biological functionality [18]. Simultaneously, emerging cross-linking approaches-based on dynamic covalent bonds and supramolecular interactions-have shown potential in reinforcing network integrity while maintaining biocompatibility [19,20]. Additionally, combining polysaccharides with synthetic polymers offers a modular framework to fine-tune the physicochemical properties of hydrogels without sacrificing their biocompatible nature.

**Scheme 1:**
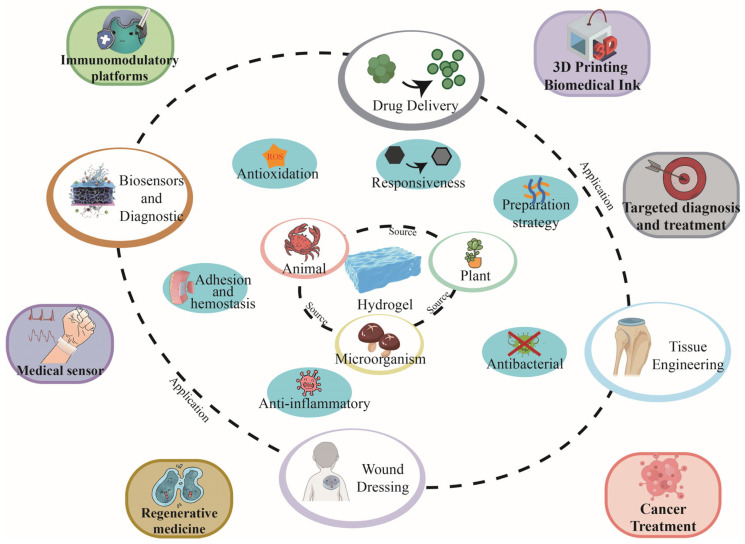
Natural polysaccharide-based hydrogels: Recent advances in biomedical applications.

Although there have been many excellent review articles that have explored the technology of polysaccharide-based hydrogels, most of these articles have focused on isolated aspects—such as concentrating on the applications on 3D dressings, or on a single source of polysaccharides, etc. The overall objective of this review is to summarize the latest advancements of natural polysaccharide hydrogels in the medical field since 2020, particularly the research conducted in the past three years. Focusing on strategies for promoting biomedical applications, this article elaborates on chemical modification, dynamic cross-linking and hybridization strategies from the perspectives of therapeutic functionality and clinical translation. In particular, it discusses the structure-performance relationships of these materials, their biomedical applications, and the challenges encountered during the translation process (Scheme 1). We discussed the latest advances in hybridization technology and new cross-linking methods, and clarified the key design principles for improving functionality. This review aims to promote the transformation of natural polysaccharide-based hydrogels into clinical applications, and to align material innovation with biomedical efficacy and environmental sustainability [21]. This perspective not only solves the limitations of transformation applications observed in previous literature, but also provides researchers with strategic guidance for selecting appropriate natural polysaccharide-based hydrogel systems, thereby significantly improving the reference value of this review.

## 2. Different Kinds of Natural Polysaccharide-Based Hydrogels

**Figure 1:**
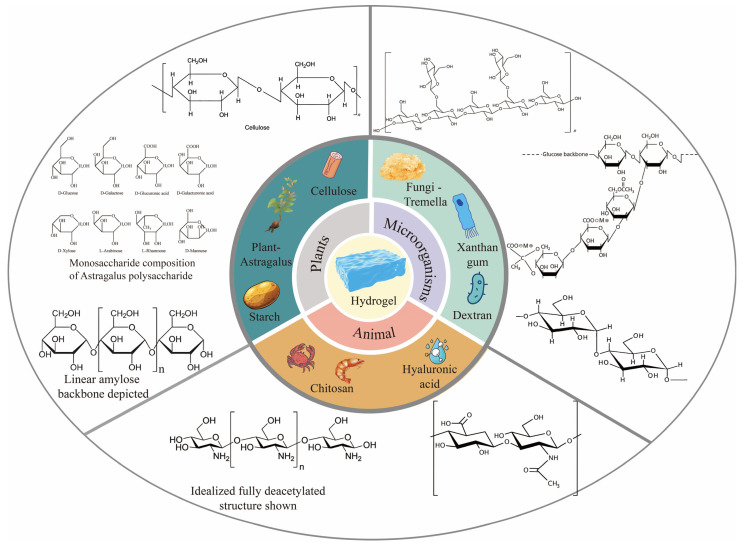
Classification and structure of common natural polysaccharides.

Natural polysaccharides can be classified into three major categories based on their biological sources: plant, animal, and microbial. This classification is mainly based on their unique tissue distribution, structural characteristics, biological activities, and extraction properties: Plant polysaccharides are widely distributed in organs such as roots, stems, leaves, flowers, and fruits, with a wide range of molecular weight distribution and complex and diverse structures. The extraction process often requires high temperatures and large amounts of solvents; Animal polysaccharides (such as hyaluronic acid, chitosan) have excellent biocompatibility with human tissues, low immunogenicity, and can be enzymatically decomposed into non-toxic small molecules. They are often found in skin, joints, and crustacean shells, and often carry specific functional groups (such as the amino groups of chitosan) that give them unique functions such as antibacterial properties; Microbial polysaccharides are synthesized by bacteria, fungi, and yeasts, and have the advantages of high production efficiency, simple extraction, uniform structure, and ease of large-scale preparation, such as xanthan gum, β-glucan, etc. At present, researchers have developed natural polysaccharides from different sources and used them in biomedical materials. This part classifies common natural polysaccharides and lists some structures (Figure 1).

### 2.1. Plant-Derived Polysaccharide Hydrogels

Plant-derived polysaccharides are widely distributed in various plant organs such as roots, stems, leaves, flowers, and fruits, and are renowned for their diverse biological activities. Zhou et al. demonstrated that *Morinda officinalis polysaccharide* can regulate the composition and metabolic activity of the intestinal microbial community, increase the relative abundance of beneficial bacteria such as *Lactobacillus* and *Bifidobacterium*, and reduce the content of potential pathogenic bacteria such as *Shigella* [22]. Plant polysaccharides also have a wide molecular weight distribution range, typically ranging from tens of thousands to millions of daltons. High-molecular-weight polysaccharides usually possess excellent gelation ability, thickening performance, and structural stability, while low-molecular-weight polysaccharides are more easily absorbed and utilized. For example, Sang found that although high-molecular-weight alginate has poor fluidity in an anhydrous system, its gel phase exhibits significant shear thinning behavior and high solid content characteristics, making it suitable for wet spinning processes [23]. Yang isolated anti-aging polysaccharides (10–50 kDa) from fermented *Panax quinquefolius*, and this substance showed significant anti-aging effects in animal models [24]. The structural complexity of plant polysaccharides also determines their diverse biological functions—even if the primary structure is the same, differences in secondary structures and tertiary conformations can lead to significantly different physiological properties. Analysis of four *Lycium species* indicated that the differences in monosaccharide composition and glycosidic bond types can result in unique thermal and chemical properties [25]. To enhance functional performance, researchers have regulated the physicochemical and biological properties of natural polysaccharides through chemical modification methods such as phosphorylation, sulfation, and methylation [26,27]. Under weak alkaline conditions, Xie et al. grafted dopamine onto sodium alginate to form a mercapto-terminal catechol group [28]. This reaction reduced the generation of oxidized catechol and enhanced the internal network structure of the hydrogel, thereby improving tissue adhesion and mechanical properties. By adjusting the mass ratio of the two precursors, the performance of the hydrogel can be further regulated. In summary, these research results fully demonstrate that plant-derived polysaccharides, as functional and multifunctional biomaterials, have great potential in the field of biomedical applications.

Although plant polysaccharides possess a wide range of biological functions, their effective utilization still faces numerous challenges, among which the extraction process is one of the main obstacles. The extraction process plays a decisive role in the yield, purity, and functional activity of the resulting hydrogel polysaccharides. Traditional extraction methods usually require high temperatures and large amounts of solvents, which can degrade heat-unstable bioactive compounds and reduce extraction efficiency [29]. To address these limitations, modern green extraction technologies have emerged, which can simultaneously improve the quality and yield of polysaccharides. For instance, the emergence of enzyme-assisted ultrasonic extraction technology has increased the extraction yield, and the extracted polysaccharides also exhibit stronger anti-lipid peroxidation activity and reducing ability [30,31]. These advancements in extraction technologies have laid the foundation for the efficient and sustainable utilization of plant-derived polysaccharides. At the same time, the progress of three-dimensional bioprinting technology has further expanded the application prospects of plant polysaccharide hydrogels [32]. Although these encouraging advancements have been made, there are still many significant challenges—especially in enhancing mechanical strength, controlling degradation rates, and improving functional adaptability in clinical applications. To overcome these limitations, recent research increasingly focuses on developing hybrid hydrogel systems, multi-responsive structure design, and scalable preparation technologies. At the same time, the integration of green processing methods and targeted biofunctionalization strategies demonstrates great potential in enhancing biocompatibility and specific application performance. These innovations collectively enhance the transformation application potential of plant-derived polysaccharide hydrogels, making them a sustainable and effective synthetic biomaterial alternative in the next generation of biomedical technologies.

### 2.2. Animal-Derived Polysaccharide Hydrogel

Animal-derived polysaccharides are natural biological macromolecules that exhibit excellent biocompatibility with human tissues, do not trigger severe immune responses, and are generally non-toxic and free of side effects [33]. These properties make them highly suitable for in vivo biomedical applications. A representative example is hyaluronic acid, a glycosaminoglycan composed of alternating D-glucuronic acid and D-glucosamine units linked via β-1,3 and β-1,4 glycosidic bonds. It is abundantly found in animal tissues such as skin, joints, and eyes. Owing to the presence of multiple hydroxyl groups in its molecular structure, hyaluronic acid is strongly hydrophilic and readily binds water molecules to form elastic, viscous hydrogels [34]. Li et al. [35] developed an acid-resistant hydrogel using hyaluronic acid, incorporating ginsenosides for the treatment of gastric wounds. The resulting hydrogel exhibited excellent hemostatic and acid-resistant properties, promoting wound healing in the gastric environment. In addition to their biocompatibility, animal-derived polysaccharides are enzymatically degradable and metabolized into non-toxic small molecules, thereby minimizing environmental persistence and pollution.

Beyond biocompatibility and degradability, animal-derived polysaccharide hydrogels often possess unique biofunctionalities. For instance, chitosan, derived from crustacean shells, contains abundant amino groups that confer a positive charge, endowing it with intrinsic antibacterial properties [36]. Chitosan gelation is highly sensitive to environmental pH. It is insoluble in neutral water but becomes soluble under acidic conditions due to amino group protonation. Upon neutralization (pH > 6.5), protonated amino groups lose their charge, resulting in rapid hydrogel formation [37]. Ionic interactions also contribute to gelation, such as interactions between chitosan residues and molybdate polyoxyanions under acidic conditions [38]. Given chitosan’s limited solubility in neutral or alkaline media, various chemical modifications have been explored to improve its solubility, targeting ability, and therapeutic performance. Derivatives such as carboxymethyl chitosan, methacrylated chitosan, thiolated chitosan, and ethylene glycol chitosan have demonstrated improved pharmacokinetics and biofunctionality [39]. For example, Firuzeh et al. developed a bilayer multifunctional hydrogel composed of quaternary ammonium chitosan, β-glucan, and curcumin, which exhibited strong adhesion to HaCaT cells, enhanced wound healing, and broad-spectrum antibacterial activity [40]. The incorporation of quaternary ammonium groups significantly improved chitosan’s hydrophilicity and antibacterial efficacy across a wide pH range.

### 2.3. Microbial Polysaccharide Hydrogels

Polysaccharides derived from microbial sources, which are biopolymers synthesized by bacteria, fungi, and yeasts, possess advantages such as high production efficiency, easy extraction, and uniform structure, making them ideal materials for large-scale application in biomedicine. Trombino encapsulated omega-PUFA using the antioxidant property of xanthan gum, significantly inhibiting the proliferation of colorectal cancer cells in vitro [41]. However, the yield based on direct secretion collection is often unstable. To address this issue, researchers have improved the extraction method. Yan employed the ultrasonic-assisted enzymatic extraction method (UAE) to isolate polysaccharides rich in β-glucan from yeast spores, achieving a sugar content of 5.964% under optimized conditions [42]. Different extraction methods may have significant effects on the activity of polysaccharides. Li used a scanning electron microscope (SEM) to compare the extraction effects of ultrasonic-assisted enzyme extraction (UAE) and microwave-assisted enzyme extraction (MAE) on the polysaccharides in *Rhodomyrtus tomentosa* berries, revealing their unique antioxidant properties and structural characteristics. The ABTS radical scavenging ability of UAE was significantly higher than that of MAE [43].

Among microbial polysaccharides, hyaluronic acid and xanthan gum have been studied in depth in promoting cell adhesion, proliferation, and extracellular matrix remodeling [44]; the polysaccharides composed of α-1,6-linked glucose units have excellent solubility and are widely used in injectable hydrogels, wound dressings, and drug carriers [45]. Chen studied β-glucan from *Hericium erinaceus*, which can self-assemble into a stable helical conformation through freeze–thaw cycles and solvent exchange, achieving this process without chemical modification [46,47]. Salecan is a water-soluble β-glucan secreted by Agrobacterium ZX09, which has been used to develop hydrogels with controllable mechanical properties and biological activity. Wang developed a hydrogel based on Salecan through free radical polymerization technology, enabling pH-sensitive and dose-dependent polyhexamethylene biguanide release [48]. Additionally, in another study on the drug encapsulation and release of gellan gum, H. Mahmood used the solvent casting ion gelation method to encapsulate ofloxacin and tea tree oil or lavender oil in gellan gum hydrogel membranes [49]. The prepared membrane material is transparent and flexible, possessing antioxidant activity and antibacterial properties, and can be used for treating full-thickness wounds.

Microbial polysaccharide hydrogels are gradually being applied in the fields of wearable and implantable biomedical devices due to their ionic conductivity, flexibility, and tissue compatibility [50]. In particular, Qing et al. developed an ionically conductive hydrogel composed of PVA, xylan polysaccharide, and sodium chloride through freeze–thaw processing and salt immersion methods [51]. This hydrogel exhibits high mechanical strength (2.72 MPa), strong ionic conductivity (10.44 S/m), and a modulus factor of 5.98, enabling its integration into wearable strain sensors that can monitor joint movements in real time. With its sustainable source, controllable structural characteristics, and extensive functional potential, microbial polysaccharide-derived hydrogels are expected to play a role in the next generation of medical materials.

## 3. Fabrication Techniques

**Figure 2:**
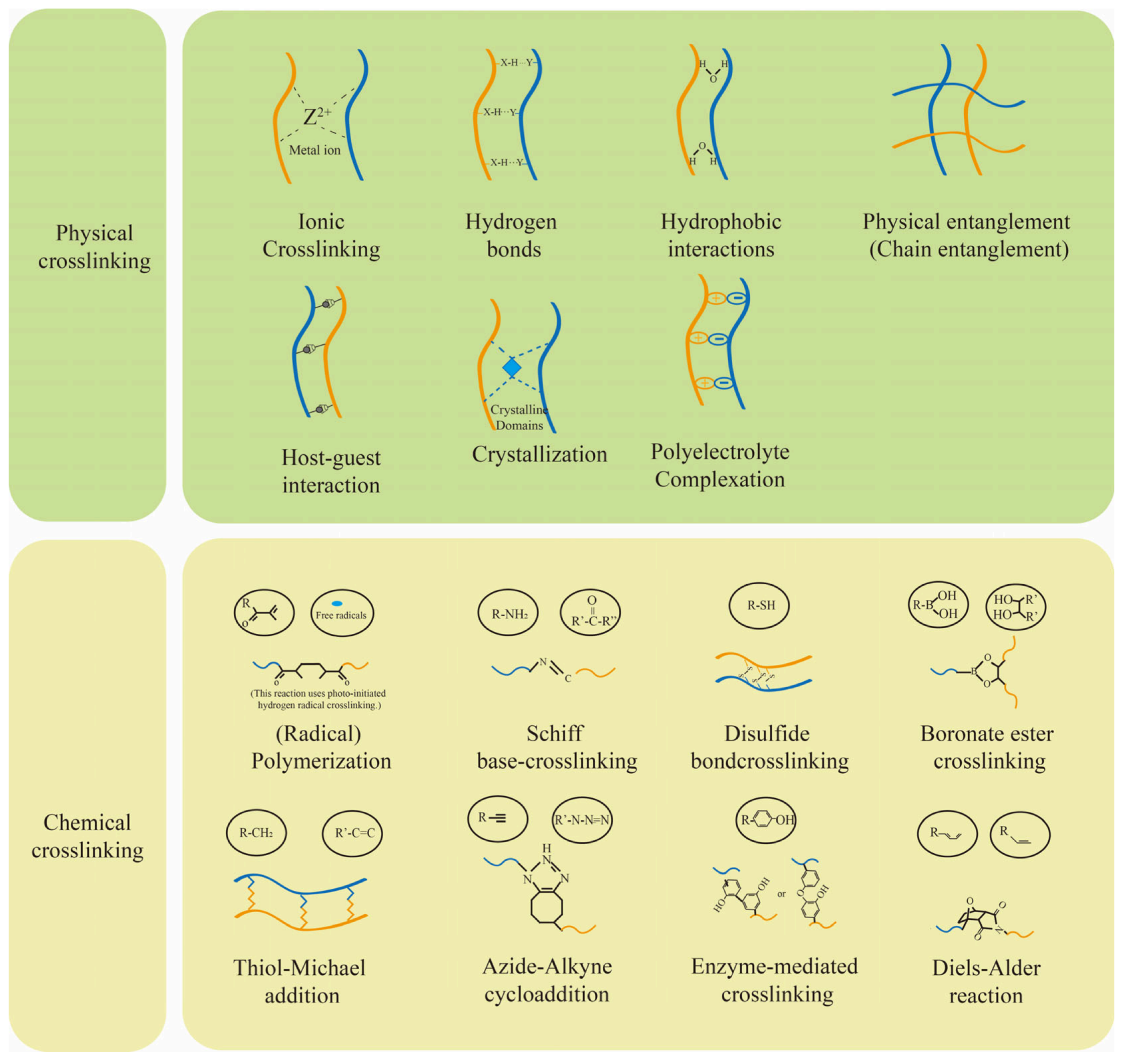
Schematic illustration of chemical and physical cross-linking strategies used in the design and fabrication of polysaccharide-based hydrogels. Yellow and blue represent different long chains of polysaccharides.

Polysaccharide-based hydrogels are highly regarded for their excellent biocompatibility and biodegradability, and are ideal candidates for biomedical applications. The key link in the preparation process is the crosslinking technology, which directly determines the formation of a stable three-dimensional polymer network. The cross-linking strategy can be roughly divided into two categories: chemical method and physical method. Each method has a unique mechanism of action, and both methods can realize the regulation of specific performance parameters such as stiffness, viscoelasticity and responsiveness. This section will summarize the physical and chemical crosslinking methods (Figure 2), and compare and analyze their respective advantages and disadvantages from the perspective of hydrogel performance and application potential (Table 1).

### 3.1. Physical Cross-Linking

Physical crosslinking refers to the formation of reversible networks through non-covalent interactions such as hydrogen bonds, electrostatic forces, and hydrophobic forces. This method typically does not require the use of chemical crosslinking agents, thus preserving the natural structure and biological activity of polysaccharides. Due to the abundance of hydroxyl groups and other functional groups, polysaccharide molecules can easily form hydrogen bonds, thereby forming a physical entangled network [52]. Zhao et al. observed reversible intramolecular interactions in branched starch/polyacrylamide hydrogels: the continuous hydrogen bond network endowed it with excellent mechanical extensibility. These hydrogels can be stretched from less than 0.5 cm to over 300 cm without breaking, with an elongation rate exceeding 36,000%, and an ultra-high pressure strain capacity of 22,500%, while also possessing excellent toughness (47 MJ·m^−3^) [53]. These properties make them ideal candidate materials for the field of human motion perception and energy storage, thereby promoting the development process of ultra-elastic multifunctional hydrogels.

Polysaccharide molecules can also spontaneously self-assemble into ordered supramolecular structures under specific conditions, forming fibrous, microsphere-like, or other network structures through hydrogen bonds and hydrophobic interactions [54]. Zhu isolated polysaccharides from the well-known Chinese herb *Polygonatum sibiricum* Red. (Huangjing) and combined them with chitosan (CS) to prepare PSP/CS hydrogels, forming a three-dimensional network of porous structure composed of nanofiber chains. The study demonstrated that hydrogen bonds were the main driving force for self-assembly in this system. As the concentration of polysaccharides increased, the gel strength and stability significantly improved. Additionally, the PSP/CS hydrogels exhibited a high water absorption expansion rate (2673 ± 65%) and water retention capacity (78.26 ± 1.58%) [55]. This provides a new method for designing nanofiber hydrogels using natural bioactive compounds for chronic wound treatment. Physical crosslinked hydrogels can also gain advantages through the synergistic action of multiple dynamic bonds. WANG et al. [56] designed an intelligent hydrogel that responds to acidic pH, reactive oxygen species, and high glucose levels. Its crosslinking network is based on dynamic acylhydrazone bonds and phenylboronic acid ester bonds. In the diabetic wound microenvironment, it undergoes specific hydrolysis and competitive reactions, achieving programmed release of tannic acid, thereby achieving multiple therapeutic purposes such as eliminating reactive oxygen species, antibacterial, promoting angiogenesis, and extracellular matrix deposition. Designing multifunctional, stimulus-responsive, and intelligent hydrogel systems is also an important direction.

### 3.2. Chemical Cross-Linking

Chemical crosslinking involves the formation of irreversible covalent bonds between polymer chains, usually requiring an external crosslinking agent. Some chemical crosslinking systems are simple, such as the hydrogel based on gum acacia-grafted-poly was prepared using ammonium persulfate (APS) as the initiator and N,N’-methylbisacrylamide as the crosslinking agent [57]. Hussain synthesized a semi-interpenetrating hydrogel network by grafting polyacrylamide onto almond gum and introducing silver ions to achieve comparable antibacterial activity to ampicillin. However, traditional crosslinking agents like glutaraldehyde have high cytotoxicity and a risk of calcification [58]. Therefore, low-toxic or natural crosslinking agents are increasingly favored. Natural compounds such as genipin and polyphenols not only enhance mechanical strength but also improve biocompatibility and environmental safety [59].

Other chemical crosslinking methods include enzyme catalysis, ionic coordination, and interpenetrating network structures [60,61]. Enzyme-mediated crosslinking technology has advantages of precise site selectivity and environmental friendliness. In Rahvar’s research, a hybrid hydrogel with different HA-Tyr/COL-II-Tyr ratios was prepared using horseradish peroxidase and a non-cellularly toxic concentration of hydrogen peroxide for encapsulating human bone marrow-derived mesenchymal stromal cells (hBM-MSCs) [62]. This method provided a more favorable microenvironment for the chondrogenic differentiation of hBM-MSCs. Ion crosslinking (usually involving metal ions such as Fe^3+^) can significantly enhance the mechanical properties of the hydrogel. Chemical crosslinking strategies are continuously evolving towards directions from biocatalysis, to efficient chemical selectivity, to precise and spatially controllable control.

**Figure 3:**
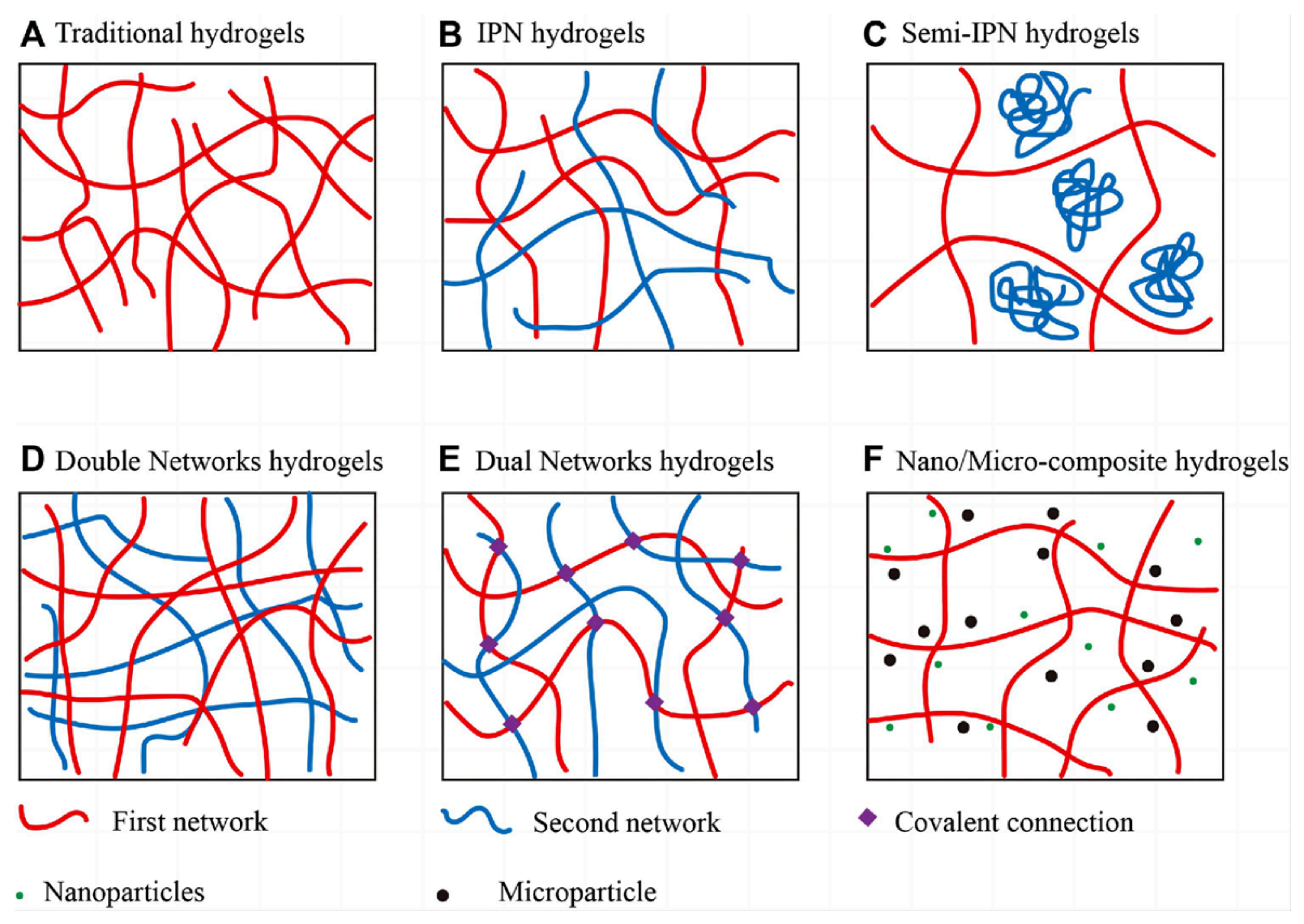
Schematic illustration of various structural forms of injectable hydrogels. (**A**) Traditional single-polymer network. (**B**–**F**) Diverse multiscale structures of injectable hydrogels, including interpenetrating polymer networks (IPNs) and other composite architectures [65]. Copyright 2022 Elsevier.

Crosslinking strategies are not always limited to a single method (Figure 3); the rational design of multiple crosslinking strategies can enable hydrogels to possess the desired specific properties [63]. Traditional hydrogels are composed of a single polymer network and have relatively weak mechanical properties; IPN hydrogels are formed by the physical interpenetration of two independent cross-linked networks, which enhance strength and stability through synergy; Semi-IPN hydrogels contain only one cross-linked network, with the other component interpenetrating in a linear chain form, thus being more flexible and responsive; Double Networks hydrogels are also interpenetrated by two cross-linked networks, but usually adopt the strategy of “rigid brittle network + flexible network”, achieving ultra-high toughness through the energy dissipation mechanism of the first network; Dual Networks hydrogels introduce covalent bonds to connect the two networks, chemically coupling them and making the structural integrity stronger; Nano/Micro-composite hydrogels achieve multi-scale enhancement by dispersing nanoparticles or microparticles in the polymer matrix, and endow them with additional functions such as conductivity and antibacterial properties. Tavakoli uses electron beam (e-beam) cross-linking as a clean and additive-free process. It is an extensible green cross-linking process that avoids the toxic chemical additives required by traditional chemical cross-linking agents, demonstrating a practical, stable, lightweight and efficient polymer hydrogel platform [64]. This material not only exhibits excellent cell compatibility but also shows great potential in tissue engineering applications. Particularly noteworthy is the integration of green crosslinking strategies, which provides new ideas for improving biocompatibility, promoting clinical translation to achieve sustainable biomedical solutions.

## 4. Application of Polysaccharide-Based Hydrogels

To thoroughly investigate the potential applications of polysaccharide-based hydrogels, this section presents a systematic review of recent research advancements in the field (Table 2). Given the promising prospects of these materials, the discussion is focused on four key areas: Tissue Engineering and Regenerative Medicine, Drug Delivery, Biosensors and Diagnostics.

**Table 1:** Cross-linking strategy of natural polysaccharide hydrogels.

Cross-Linking Method	Component Parts	Structural Type	Crosslinking Method or Crosslinking Agent	Advantages	Ref.
Chemical crosslinking	Almond gum	Semi-IPN	N,N′–methylenebisacrylamide	A semi-interpenetrating network is formed through free radical polymerization initiated by REDOX reactions	[59]
	whey protein isolate/chitosan	Traditional	genipin	It begins to form a gel upon mixing at room temperature, maintaining a high compressive strength while also maintaining a high water retention rate	[66]
	borated peach gum and oxime-modified hyaluronic acid.	Double Networks	arginine	Arginine, as a cross-linking agent, further enhanced its biocompatibility and functional performance.	[67]
	phenylboronic acid-modified marine-derived sodium alginate	Nano/Micro-composite	gold clusterzyme	It greatly enhances the mechanical properties of the hydrogel, endows the hydrogel with good tissue adhesion, thereby achieving rapid hemostasis, and the crosslinking agent is easy to remove	[68]
	Glucan—hyaluronic acid	IPN	Horseradish peroxidase	The reaction conditions are mild, and the mechanical properties can be programmed with horseradish or oxidase/H_2_O_2_ ratio	[69]
Physical crosslinking	Amylopectin/polyacrylamide	IPN	A continuous hydrogen bond network	It features extremely high deformation, capable of stretching from less than 0.5 to over 300 cm without breaking, with an elongation rate exceeding 600 times the original length.	[53]
	Chitosan/alginate	Double Networks	Hydrogen bond, double-ion crosslinking	Hydrogen bonds promote the self-crosslinking of chitosan to produce phase separation, and the first network is constructed in the hydrogel. Dual-ion crosslinking ensures the stability of alginate aggregates and constructs a second network in the hydrogel.	[70]
	Gastrodia elata polysaccharides/methacrylic acid gelatin	Dual Networks	Schiff base bonds, photo-crosslinking and hydrogen bonds	The unique multi-cross-linked design not only endows the hydrogel with excellent mechanical properties, but also provides it with rapid antibacterial, antioxidant and hemostatic capabilities.	[71]
	Hyaluronic acid/chitosan	Traditional	Hydrazone bonds, disulfide bonds, electrostatic interactions	The formation of different types of reversible interactions enables hyaluronic acid hydrogels to rapidly gel and exhibit excellent self-healing capabilities, achieving complete healing within one hour.	[72]
	Tamarind seed polysaccharide and sodium alginate	Double Networks	Physical cross-linking and enzymatic covalent cross-linking	By leveraging the complementary synergy of two networks with different properties, we can overcome the performance limitations of a single network.	[73]

**Table 2:** Application potential and advantages of natural polysaccharides.

Polysaccharide	Source	Functional Groups	Advantages	Potential Properties	Ref.
Tissue engineering and regenerative medicine	Hyaluronic acid	-OH; -COOH	It overcomes the rejection reaction of cells to polysaccharide-based scaffolds, promotes cell adhesion and aggregation, and enhances the interaction between cells and cytosol.	Regulate the immune microenvironment and create a regenerative microenvironment.	[72]
	Hydroxyethyl starch	-OH; -CHO	The mechanical properties can be adjusted by regulating the aldehyde-amine ratio.	It can be used for soft tissue adhesion, hemostasis and wound healing	[74]
	Chitosan	-OH; -NH_2_	Abundant hydroxyl groups form strong hydrogen bonds with water molecules, resulting in a very high water content	It can promote angiogenesis and collagen deposition, minimizing scar formation to the greatest extent.	[75]
	Astragalus polysaccharides	-OH	The mechanical span characteristics of continuous gradient hydrogels have been achieved.	It is a bionic scaffold with improved mechanical span, which closely replicates the mechanical heterogeneity of natural osteochondral tissue	[76]
Drug delivery	Alginate	-COOH; -OH; -CHO	The drug is dispersed in the heat-responsive hydrogel and ROS reactive drug release is achieved.	It can cross the blood–brain barrier and deliver drug components to the brain.	[77]
	Chitosan	-OH; -NH_2_	The hydrogel exhibits self-repairing properties, showing antioxidant and antibacterial properties in response to ph-drug delivery.	It is a biocompatible and stimulus-reactive drug carrier	[78]
	Dioscorea opposita Thunb polysaccharide	-OH; -COOH	It can prevent the leakage of loaded drugs and effectively generate the release triggered by glucose	It can be used as an “intelligent” glucose response carrier to control the slow release of drugs.	[79]
Wound Dressing	salecan	-OH	Hydrogels exhibit self-healing, high adhesion and repairable behaviors	This hydrogel promotes the exchange of nutrients, regulates oxygen permeability, and absorbs wound exudate through its porous matrix	[80]
	Chitosan	-OH; -NH_2_	Through the synergistic antibacterial effect of photothermal activation and metal ion coordination	It has achieved a bactericidal effect of over 99% against multi-drug resistant (MDR) bacteria.	[81]
	Chitosan	-OH; -NH_2_	After absorbing water, it exhibits electrical conductivity and network remodeling properties, and also shows degradability, rapid swelling, excellent antioxidant, adhesive, biocompatibility, photothermal and antibacterial properties.	The compound cryogel hemostatic dressing is used to stop bleeding and further promote wound healing	[82]
	Alginate	COOH; -OH	It has anti-inflammatory and pain-relieving activities and is used to treat infectious burn wounds	It not only supports wound closure but also minimizes the risk of infection and complications to the greatest extent	[83]
Biosensors and diagnostics	Tremella aurantialba polysaccharide	-OH; -COOH; -CHO	Hydrogels possess excellent transparency, thermoplasticity and remarkable mechanical properties, including significant elongation and high self-healing rate	It can accurately monitor different human movements through strain sensors. Meanwhile, it maintains excellent sensing stability and durability under repeated strain cycles.	[84]
	Bletilla striata polysaccharides	-OH	It is a heat-sensitive hemostatic hydrogel that achieves hemostasis within 30 s	It is conducive to the development of minimally invasive hemostasis in vivo and functional hemostatic gels	[85]
	Sanghuang polysaccharides	-OH; COOH	The obtained hydrogel has excellent self-healing performance. Meanwhile, SHP endows the hydrogel with antibacterial, anti-inflammatory, ROS clearance and pro-angiogenic functions, and low pH and near-infrared (NIR) irradiation can accelerate the release of sanghuang polysaccharides	Hydrogels accelerate wound healing by enhancing M2 polarization, promoting angiogenesis and reducing inflammation.	[86]
	Hyaluronic acid	-OH; -COOH	Significantly increase cell proliferation and improve wound closure	Due to the electroosmotic phenomenon and the amplification and transfer of soluble growth factors, the cell activity was significantly enhanced	[87]

### 4.1. Tissue Engineering and Regenerative Medicine

**Figure 4:**
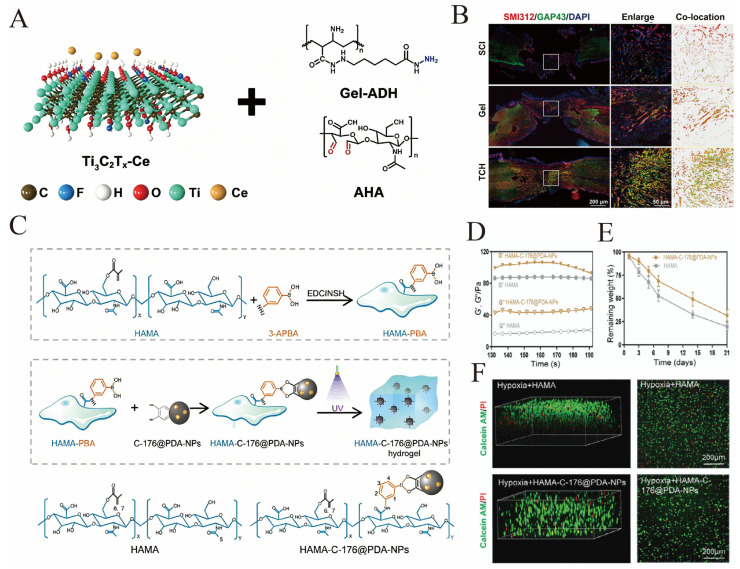
(**A**) Schematic diagram of the preparation of Ti_3_C_2_Tx-Ce conductive hydrogel. (**B**) Localization of SMI312 and GAP43 indicates that nerve axons are regenerating after spinal cord injury [90]. Copyright 2026 Wiley. (**C**) Flowchart of the preparation process of HAMA and HAMA-C-176@PDA-NPs. (**D**) Rheological properties of HAMA and HAMA-C-176@PDA-NPs hydrogels. (**E**) Degradation curves of HAMA and HAMA-C-176@PDA-NPs hydrogels. (**F**) Live/dead staining and analysis of SSCs in HAMA and HAMA-C-176@PDA-NPs hydrogels [93]. Copyright 2025 Wiley.

The development of biological scaffolds plays a crucial role in tissue engineering. Natural polysaccharide hydrogels closely mimic the extracellular matrix (ECM) due to their highly hydrated three-dimensional networks, making them particularly attractive for tissue engineering applications [88]. Gan et al. constructed a biomimetic extracellular matrix (ECM) scaffold by integrating polydopamine-modified hyaluronic acid hydrogel into a double-crosslinked collagen matrix [74]. This composite hydrogel overcomes the limitations of negatively charged polysaccharide-based scaffolds, such as poor cell adhesion. By enhancing cell aggregation and cell–matrix interactions, this scaffold significantly promotes cartilage differentiation. Moreover, such hydrogels can regulate the local immune microenvironment and promote the formation of a regenerative microenvironment that supports cartilage repair. In soft tissue engineering, adhesive hydrogels that can adapt and adhere to moist biological surfaces are essential [89]. Liu et al. designed an aldehyde-functionalized hydroxyethyl starch (AHES) using peroxide oxidation and subsequently crosslinked it with amino-functionalized carboxymethyl chitosan (ACC) through a Schiff base reaction [75]. This in situ formed hydrogel can achieve tunable mechanical properties by adjusting the ratio of aldehyde to amine, making it suitable for tissue adhesion, hemostasis, and wound healing applications. In the field of nerve repair, extensive neuronal death, mitochondrial dysfunction, and an inhibitory reactive oxygen species (ROS) enriched microenvironment are obstacles to treatment. The Schiff base formation gel composed of oxidized hyaluronic acid (AHA) and dimethylenephosphonate-modified gelatin (Gel-ADH) encapsulates and protects neurons [90]. Conductive Ti_3_C_2_Tx-Ce nanosheets reduce mitochondrial ROS and restore ATP production (Figure 4A,B), alleviating mitochondrial dysfunction and promoting neuronal electrical activity and dependent Ca^2+^ influx.

For bone tissue engineering, hydrogels must have sufficient mechanical strength to support structural integrity and promote bone regeneration. Injectable polysaccharide-based hydrogels are particularly attractive because they have characteristics similar to the extracellular matrix, high water content, and the ability to deliver bioactive agents in a controlled manner. Despite significant progress, most polysaccharide hydrogels still exhibit inadequate mechanical properties for load-bearing tissues such as cartilage and bone [91]. To address this limitation, Xu et al. developed a novel continuous magnetic gradient hydrogel inspired by the biomechanical heterogeneity of natural bone-cartilage tissue [92]. Glycyrrhizin polysaccharide was incorporated into the magnetic-responsive matrix, resulting in a compressive modulus gradient from 17.1 kPa at the surface to 419 kPa at the base—approximately 24.5 times the span. When combined with an external magnetic field, this gradient hydrogel significantly enhanced the repair effect of bone-cartilage defects. At the 12th week after implantation, the treated joint showed complete recovery and a smooth articular surface. This strategy provides a scaffold with mechanical and functional gradients inspired by natural tissue structure, which is very close to the natural tissue structure. To some extent, for complex tissue regeneration similar to bone tissue, hydrogels not only need to have extensibility but also need to have certain toughness. Here, Luo developed a 3D-printed Ti6Al4V scaffold conformally coated with photocrosslinked hyaluronic acid hydrogel (Figure 4C–F) that retains and releases Piezo1-engineered exosomes (P-Exos), forming a hybrid construct with cancellous-bone-like stiffness and sustained bioactivity [93]. The plasticity of 3D printing can be applied to other special applications, such as tubular organs. Archana developed an antibacterial catheter using pressure-assisted 3D printing technology [94]. This catheter was filled with cinidazole, which can disrupt bacterial biofilms and inhibit key pathogenic factors mediated by sensory proteins that cause urinary tract infections. This strategy is expected to be applied in biomedical fields, including the regeneration of complex tissues and organs.

Future research should focus on developing intelligent hydrogels with biomimetic gradient structures and dynamic regulation capabilities to more accurately simulate the microenvironment of natural tissues. By integrating 3D/4D bioprinting, stem cell technology, and exosome delivery systems, it is expected to achieve precise regeneration of complex tissues and organs. At the same time, enhancing the in vivo repair effect through immune regulation and vascularization design will become an important development direction for tissue engineering hydrogels.

### 4.2. Drug Delivery

**Figure 5:**
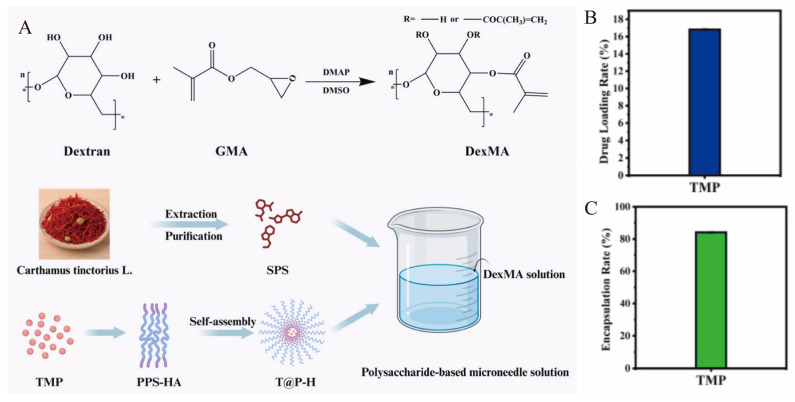
(**A**) Schematic diagram of D/SPS/T@P-H. “*” indicates the omitted part of the dextran. (**B**) Drug loading of T@P-H (**C**) Encapsulation rate of T@P-H [95]. Copyright 2026 Elsevier.

Natural polysaccharide hydrogels possess excellent drug encapsulation capacity and sustained-release properties, which can effectively protect drugs from degradation in the body, improve drug stability and bioavailability. Their network structure is easily adjustable and enables the delivery of small molecule drugs, proteins, growth factors, and nucleic acid drugs. Moreover, polysaccharide materials have good biological safety and injectability, making them ideal drug delivery carriers. Xu et al. designed a natural polysaccharide-based nanogel modified with transferrin and disulfide bonds [77]. Zhong [95] developed a hydrogel matrix of methylacrylated dextran (DexMA), which served as both an oxidoreduction-sensitive carrier and a hydrophilic polysaccharide component, and synergistically delivered tetramethylpyrazine (TMP) and safflower polysaccharide (SPS), accelerating wound healing in diabetic rats (Figure 5). The disulfide linkages endowed the system with reactive oxygen species (ROS)-responsive characteristics, allowing the nanogel to cross the blood–brain barrier and co-deliver two traditional Chinese medicine (TCM) components, glycyrrhizic acid and paeoniflorin, to the brain. Upon administration, the concentrations of glycyrrhizic acid and paeoniflorin in rat hippocampal tissue were increased by approximately three-fold and five-fold, respectively, compared to the drug concentrations delivered by conventional hydrogel carriers. Meanwhile, the ROS concentration in the hippocampus decreased from 200 ng/mL to 100 ng/mL confirming the system’s therapeutic efficacy and oxidative stress mitigation potential.

In recent years, stimulus-responsive polysaccharide hydrogels have become a research hotspot. By introducing pH-responsive, temperature-responsive, ROS-responsive and glucose-responsive structures, precise drug release can be achieved upon triggering by the microenvironment of diseases. For instance, Jeong et al. synthesized a pH-sensitive hydrogel via imine bonds and ionic interactions between oxidized hydroxybutanoyl glycan (OHbG) and quaternized carboxymethyl chitosan (QCMCS) [78]. Due to the inherent instability of imine bonds, the hydrogel exhibited distinct pH-responsive release behavior, with drug release reaching 96.57% at pH 2.0 and 63.22% at pH 7.4. The presence of quaternary ammonium groups in QCMCS also conferred strong antibacterial activity to the hydrogel. In another study, Liu et al. investigated the chemical cross-linking between yam-derived polysaccharides and borate ester bonds [79]. Under high-glucose conditions, the release of yam polysaccharides and the co-delivered compound trans-N-p-coumaroyltyramine (NCT) significantly alleviated insulin resistance. The hydrogel promoted glucose consumption in insulin-resistant HepG2 cells, increasing utilization rates to 65%. At present, the polysaccharide hydrogel drug delivery system still faces problems such as rapid drug release, limited drug loading efficiency, and insufficient long-term stability. The drug release behavior in complex disease environments is difficult to be precisely controlled, and there is still a lack of systematic research on the in vivo metabolism and pharmacokinetic mechanisms. Moreover, there is still a significant gap between laboratory research and clinical translation. In the future, efforts should be focused on developing multi-stimulus-responsive and intelligent feedback-based drug delivery systems to achieve more precise and controllable drug release at specific times and locations. By integrating nanotechnology, targeted ligands, and artificial intelligence-assisted design, the treatment accuracy and individualized level can be further enhanced. At the same time, strengthening in vivo behavior research and clinical validation will drive the development of polysaccharide hydrogels towards precision medicine.

### 4.3. Wound Dressing

**Figure 6:**
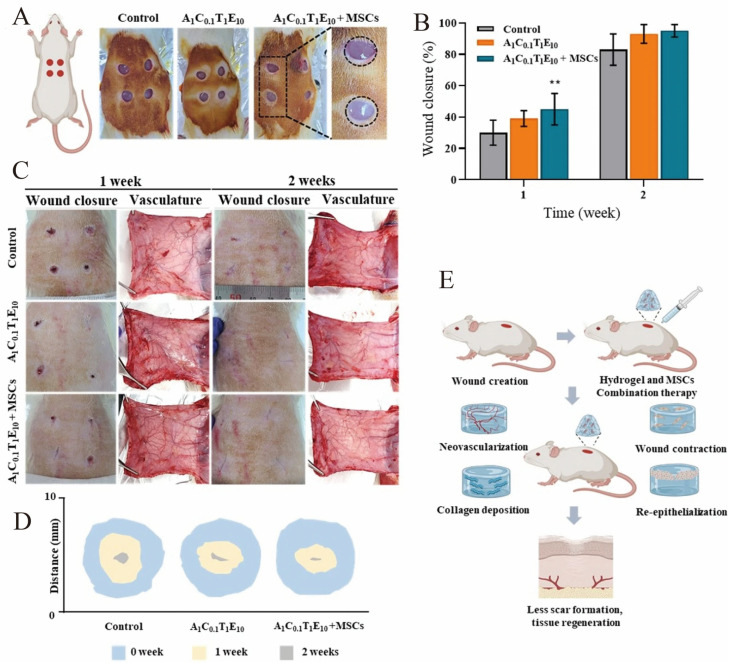
In vivo evaluations of the wound healing process (**A**) Photograph of the rat model of excisional wounds during application (**B**) Percent wound closure of control and after applying sample for cutaneous wound model (**C**) Photographs of wound healing and vasculature after 1 week and 2 weeks (**D**) Comparative representation of the wound healing process between different groups and (**E**) Schematic illustration of wound, hydrogel application and process during wound closure. ** represents *p* < 0.01, a highly significant difference, and * represents 0.01 < *p* < 0.05, a significant difference [97]. Copyright 2026 Springer nature.

Wound healing is a complex and dynamic biological process that involves hemostasis, inflammation, cell proliferation, re-epithelialization, and tissue remodeling. Hydrogels with dynamic cross-linking, electroactivity, and antibacterial properties can significantly enhance hemostasis and wound closure efficacy in the early stages of healing [96]. Polysaccharide hydrogel has a high water content and excellent permeability, which can maintain a moist healing environment and promote cell migration and tissue regeneration. Its three-dimensional network structure can absorb exudate from the wound surface and at the same time prevent external contamination [93]. The porous structure of hydrogels facilitates nutrient exchange, regulates oxygen permeability, and absorbs wound exudates—all of which are key factors in promoting wound healing. In recent years, multifunctional wound dressings have experienced rapid development. Researchers have incorporated metal ions, nanoparticles, photothermal materials, growth factors, and stem cells into polysaccharide hydrogels to achieve antibacterial, hemostatic, anti-inflammatory, and angiogenic functions. Chen et al. addressed this issue by designing a hydrogel system that is cross-linked through dynamic Schiff base bonds and adding quaternized chitosan, copper ions, and 808-nanometer near-infrared (NIR) irradiation [81]. This composite hydrogel achieved a 99% bactericidal efficiency against multidrug-resistant (MDR) bacteria through a photothermal-metal ion synergistic mechanism. Myeongki Park [97] demonstrated that by incorporating stem cells (bone marrow-derived mesenchymal stem cells, MSCs) into an injectable wound healing agent composed of alginate, carrageenan, oxidized cellulose nanofibers (TOCN), and natural porcine skin dSECM (A1C0.1T1E10 + MSCs), the gel containing the stem cells promoted cell proliferation, ECM deposition, neovascularization, regulation of inflammatory responses, re-epithelialization, and enhancement of hair follicle and gland formation (Figure 6). This agent is suitable for severe foot ulcers in diabetic patients, pressure ulcers in the elderly and critically ill patients, as well as deep burns.

Severe skin injuries, such as burns and chronic ulcers, often exceed the regenerative capacity of the skin and are highly susceptible to bacterial infection. Normal skin typically has a weakly acidic pH, ranging from 4.5 to 6.5 [98]. This acidic environment is maintained by the skin’s “acid mantle,” which ensures barrier function, antibacterial defense, and microecological balance. After skin damage, in the early stages, due to tissue necrosis, inflammatory response, and exudation, the pH value often rises (slightly alkaline, approximately 7.7–8.2). As the wound healing process progresses, the pH value gradually decreases and tends to be weakly acidic (approximately between 6.0–7.3). However, when pathogenic bacteria infect the wound, the microenvironment of the wound is altered. Enzymes in the bacteria break down urea to produce ammonia, making the environment alkaline. Proteins in inflammatory exudates, such as albumin, increase. Local hypoxia and the accumulation of necrotic tissue also promote alkalization. The pH value on the wound surface typically rises and tends to be alkaline (7.3–9.8). This leads to an increased risk of protein denaturation, delayed epithelial tissue healing, and scar formation [99,100]. To address these challenges, hydrogels with intrinsic antibacterial and regenerative properties have been developed. For example, Deng et al. fabricated a bioactive hydrogel dressing containing analgesics and anti-inflammatory agents, with components including glycosylated grafted polyvinyl alcohol, 3-aminobenzoic acid chemically modified alginate sodium, zinc ions, and chitosan-coated camphor nanoparticles [80,83]. At present, the current wound hydrogels still face problems such as insufficient mechanical strength, limited long-term antibacterial effect, and poor adaptability to complex wound environments. For diabetic wounds and chronic wounds, the persistent infection and inflammatory responses are still difficult to be completely controlled. Moreover, the long-term storage stability and industrial production of hydrogels also need to be further optimized. In the future, wound dressings will develop towards intelligence and individualization. Intelligent hydrogels capable of real-time monitoring of infection, biomarkers, and pH changes are expected to achieve integrated diagnosis and treatment. New dressings with multiple functions such as antibacterial, antioxidant, immune regulation, and angiogenesis promotion will further enhance the therapeutic effect and clinical application value of complex wounds.

### 4.4. Biosensors and Diagnostic

Due to the advancements in materials science and biomedical fields, personalized and responsive medical solutions are currently driving the progress in the medical field [101]. Han et al. developed a polyvinyl alcohol (PVA)-based hydrogel by cross-linking larch polysaccharide and silk fibers with boric acid [83]. Due to the dynamic hydrogen bond interactions and boric acid cross-linking, the resulting hydrogel has excellent transparency, thermoplasticity, and significant mechanical properties, including an elongation rate of 1107.3% and a 91.11% self-healing efficiency within 5 min. This hydrogel can reliably monitor human movement through strain sensing and maintain stable and durable sensing performance under repeated mechanical cycles. Such intelligent hydrogels have great potential in biomedical applications. Thermosensitive hydrogels can rapidly form gels under physiological temperature conditions, allowing the gel to solidify in the lumen after injection and be used in hemostasis and tissue repair, etc. [102]. Yan et al. developed a thermosensitive hydrogel based on collagen and dendritic polysaccharide, achieving hemostatic effects within 30 s [84], laying a solid foundation for minimally invasive hemostasis therapy and functional hemostatic hydrogel. Besides temperature responsiveness, thermosensitive components can also achieve near-infrared (NIR) assisted treatment. Wang et al. incorporated MXene nanosheets into a hydrogel microgel composed of dopamine-modified chondroitin sulfate and benzoylboronic acid grafted gelatin [85,86]. This hydrogel responds to low pH values and near-infrared irradiation, accelerating the release of larch polysaccharide and enhancing antibacterial, anti-inflammatory, superoxide anion scavenging, and angiogenesis effects. Ma et al. synthesized a cost-effective multifunctional hydrogel by coupling carboxymethylated Acorus tataricus polysaccharide (BSP) with dopamine [102]. The resulting hydrogel based on CBSP has a negative charge and pH responsiveness. Changes in environmental pH values trigger structural transitions, enabling continuous cathizine release and long-term antibacterial activity. This pH-responsive behavior allows for precise regulation of drug release rates and enhances therapeutic effects. Another type of ROS-responsive hydrogel is used in oxidative stress environments. This responsive stimulus is typically achieved by introducing borate ester bonds.

**Figure 7:**
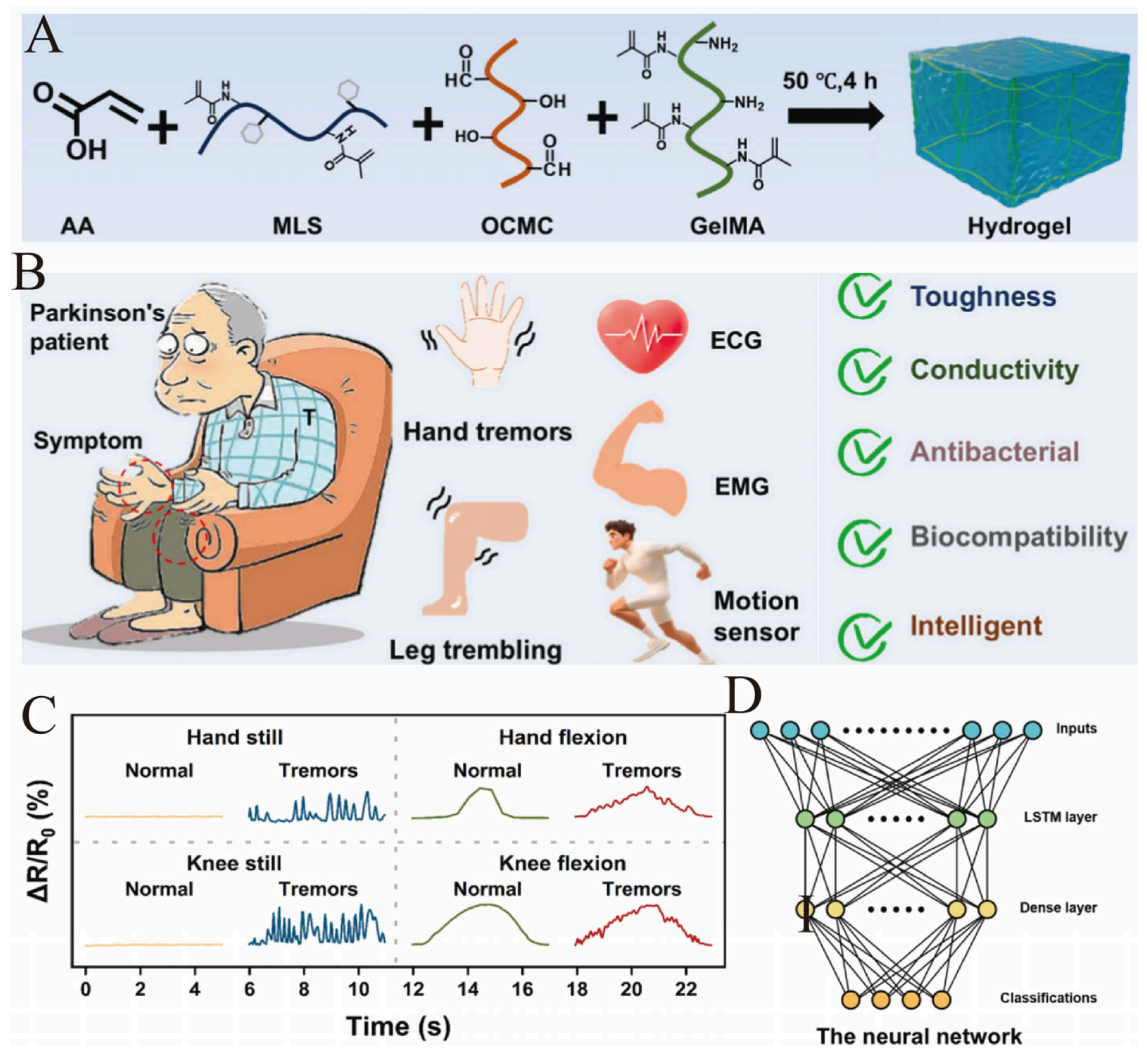
(**A**) Synthesis diagram of the corresponding components of the hydrogel. (**B**) The application and characteristics of hydrogels as sensors. (**C**) Real-time resistance monitoring across eight different body postures. (**D**) Architecture of the neural network used for symptom recognition based on resistance signals from the hydrogel sensors [104]. Copyright 2025 Elsevier.

Polysaccharide hydrogels possess excellent flexibility, extensibility and biocompatibility, and can well match human tissues. By incorporating conductive materials and dynamic crosslinking structures, they can be endowed with conductive, self-repairing and stimulus-responsive properties, thus being suitable for flexible biosensors and health monitoring devices. Chen introduced a dynamic borate ester bond cross-linking network sensitive to reactive oxygen species (ROS) and catechol groups to address bone abnormalities caused by middle ear inflammation [103]. This hydrogel can occupy and adhere to irregular tissue defects. It is not damaged by external forces and can adapt to tissue movement. A multifunctional hydrogel was prepared by a multi-component synergistic network composed of polyacrylic acid (PAA), diacetal carboxymethyl cellulose (OCMC), gelatin methacryloyl (GelMA), and methyl methacrylate lignosulfonate (MLS) [104]. It exhibited synergistically enhanced interfacial adhesion strength (>77.8 kPa) and mechanical properties, with a tensile elongation rate exceeding 1820%. The unique gradient network structure not only provided the material with excellent conductivity (conductivity > 0.55 S/m). Moreover, the electrode constructed from this hydrogel reliably recorded human electromyogram (EMG) and electrocardiogram (ECG) signals. The conductive network, combined with its wide range and high sensitivity characteristics (GF = 5.31), enables the sensor to accurately identify clinical symptoms (Figure 7), such as the characteristic tremor signals associated with Parkinson’s disease. This neural network mode consists of a Long Short-Term Memory (LSTM) layer followed by a dense classification layer. Due to the strong temporal dependence of the input signals, the LSTM layer can retain long-range dependencies, enabling the model to learn the features that change over time. The input sequence of resistance values within a fixed time interval is continuously fed into the model, thereby achieving real-time prediction.

At present, water-based gel sensors still have problems such as signal drift, insufficient long-term stability, and degradation of conductive performance. The integration between the sensors and electronic devices is still limited, and the detection accuracy and reliability in complex physiological environments need to be improved. Moreover, the biological safety of long-term implantable sensors still requires further verification. Future research will focus on the development of high-sensitivity, multimodal, and wireless bio-sensing platforms. By combining flexible electronics, artificial intelligence, and Internet of Things technologies, continuous health monitoring and early disease warning can be achieved. At the same time, intelligent water-based gel sensors with self-repairing, self-powered, and long-term stable performance are expected to become an important component of the next generation of precision medicine.

It is worth noting that 4D printing technology is an advanced manufacturing technology that introduces a temporal dimension on the basis of 3D printing. The structures printed by 4D printing can autonomously undergo shape or functional changes under external stimuli such as temperature and humidity, achieving the integration of product design, manufacturing, and assembly [105]. Crucially, the emergence of 4D printing technology combines intelligent materials with programming design, overcoming the limitations of static manufacturing, and can dynamically deform structures in real-time under specific triggering factors. This enables precise spatiotemporal control of complex and personalized medical devices at different scales, perhaps driving the strategic development of the next generation of intelligent biomedical materials.

## 5. Conclusions and Future Perspective

Polysaccharide-based hydrogels have emerged as a highly versatile and promising class of biomaterials, offering immense potential in a variety of biomedical applications, including tissue engineering, wound healing, and controlled drug delivery. Their natural abundance, low cost, excellent biocompatibility, biodegradability, and structural tunability establish a strong foundation for the development of next-generation therapeutic platforms.

Recent innovations have significantly expanded the functionality of these materials. Microbial- and plant-derived polysaccharides bearing unique chemical groups, when integrated with advanced crosslinking technologies and precise physical–chemical modifications, can greatly enhance hydrogel performance. The incorporation of stimuli-responsive elements, self-healing mechanisms, and nanostructural reinforcements has led to the development of smart hydrogels capable of dynamic responses under physiological conditions, facilitating their application in intelligent drug delivery and regenerative therapies. Furthermore, cutting-edge fabrication technologies-including 3D and 4D bioprinting-now allow the construction of personalized, architecturally complex hydrogels that adapt in real time to biological signals and mechanical forces. These advances are transforming hydrogels from passive scaffolds into active, bio-interactive systems capable of precise therapeutic interventions. Looking ahead, there are several key aspects that need to be investigated with emphasis. These include a deeper understanding of the in vivo metabolism, immunomodulatory effects and long-term biosafety of polysaccharide-based hydrogels. Bridging the gap between laboratory research and clinical implementation also needs to be overcome. Challenges in application also exist, such as regulatory standardization, scalable and cost-effective manufacturing processes, the industrial feasibility of mass production, and the need to enhance interdisciplinary collaboration among materials scientists, biomedical engineers and clinicians.

In the future, polysaccharide-based hydrogels are expected to evolve from passive biomaterials into intelligent therapeutic platforms capable of sensing, responding, and adapting to biological environments. With continued advances in biomaterial engineering, bioprinting technologies, and precision medicine, these hydrogels may play an increasingly important role in next-generation regenerative therapies and personalized healthcare.

## Data Availability

No data was used for the research described in the article.

## References

[1] Ding X., Fan L., Wang L., Zhou M., Wang Y., Zhao Y. (2023). Designing self-healing hydrogels for biomedical applications. Mater. Horiz..

[2] Cai Y., Xu D., Chong Z.E., Chen E.S.A., Gan S.W., Bok A.S.K., Lu W.F., Zhai W., Yen C.-C. (2026). Multifunctional composite microgels: From structural design to biomedical applications. Mater. Today Bio.

[3] Lin H.-F., Wang Y.-Y., Liu F.-Z., Yang Z.-W., Cui H., Hu S.-Y., Li F.-H., Pan P. (2025). Natural Bletilla striata Polysaccharide-Based Hydrogels for Accelerating Hemostasis. Gels.

[4] He C., Liu R., Bi S., Zhang L., Gu J., Yan B. (2026). Microenvironment responsive copper-polyphenol nanozyme hybrid hydrogel with NO/O2 release for healing diabetic infected wounds via immunomodulation and angiogenesis. Chem. Eng. J..

[5] Zhang Y., Dong L., Liu L., Wu Z., Pan D., Liu L. (2022). Recent Advances of Stimuli-Responsive Polysaccharide Hydrogels in Delivery Systems: A Review. J. Agric. Food Chem..

[6] Wang Y., Fu J., Zou M., Xia P., Zheng X., Huang X., Liu Y., Yang P., Liu X., Zhang D. (2026). Programming the beating heart with polymer catalysis: A therapeutic microenvironment revolution. J. Nanobiotechnol..

[7] Yang J., Yu H., Wang L., Liu J., Liu X., Hong Y., Huang Y., Ren S. (2022). Advances in adhesive hydrogels for tissue engineering. Eur. Polym. J..

[8] Qi X., Zhang Z., Xi J., Cheng L., Li Y., Yang Q., Sun Z., Zhao T., Li X. (2026). Self-assembled hydrogels based on natural active ingredients: Mechanisms, applications and characterization. J. Mater. Chem. B.

[9] Damiri F., Simińska-Stanny J., Rasoulian F., Pinal C., Kaczmarek-Szczepanska B., Patel C.D., Patel H.M., Papadimitirou S.A., Jaradat E., Paiva-Santos A.C. (2026). Emerging trends in polysaccharide-based smart PEGylated hydrogels for biomedical applications. Carbohydr. Polym..

[10] Yu W., Jin D., Zhang Y., Wang S., Yu J., Liu M., Dai Y., Yin Y., Cheng J., Liu Y. (2025). Provoking tumor disulfidptosis by single-atom nanozyme via regulating cellular energy supply and reducing power. Nat. Commun..

[11] Tang P., Xia J., Jiang S., Song H., Jin Y., Chen J., Xie D., Wu M., Li X., Lu Q. (2026). Bimetallic nanozyme-functionalized injectable hydrogel for enhanced therapy for intervertebral disc degeneration. Chem. Eng. J..

[12] Zhang M., Ren Y., Jiang X., Chen C., Wu J., Kong X. (2026). Hybrid hydrogels of polysaccharides and inorganic nanomaterials: A synergistic combat-engine for drug delivery and tissue regeneration. Mater. Today Chem..

[13] Mohanty S., Swarup J., Priya S., Jain R., Singhvi G. (2024). Exploring the potential of polysaccharide-based hybrid hydrogel systems for their biomedical and therapeutic applications: A review. Int. J. Biol. Macromol..

[14] Dong Y., Fei Y., Hu Y., Cai C., Li M., Luo Z. (2026). Microenvironment engineering with injectable hydrogel-based biofunctional scaffolds for augmenting bone defect regeneration. J. Nanobiotechnol..

[15] Dong Y., Ghasemzadeh M., Khorsandi Z., Sheibani R., Nasrollahzadeh M. (2024). Starch-based hydrogels for environmental applications: A review. Int. J. Biol. Macromol..

[16] Zöller K., To D., Bernkop-Schnürch A. (2025). Biomedical applications of functional hydrogels: Innovative developments, relevant clinical trials and advanced products. Biomaterials.

[17] Laffargue T., Moulis C., Remaud-Siméon M. (2023). Phosphorylated polysaccharides: Applications, natural abundance, and new-to-nature structures generated by chemical and enzymatic functionalisation. Biotechnol. Adv..

[18] Dixit S.S., Moorthy G. (2026). Pectin in precision medicine for targeted delivery, gene therapy, and cancer immunomodulation: A review. Int. J. Biol. Macromol..

[19] Sun B., Zhang C., Li X., Zhong L., Zhang Z., Zhou D. (2025). Nanocellulose-based hydrogels for smart sensors. Carbohydr. Polym..

[20] Liu W., Jiang Y., Shi J. (2025). Effects of selenylation on Chinese yam polysaccharides: Structure, antioxidant, and digestive properties. Food Chem. X.

[21] Liao X., Lu J., Yao J., Li J., Chen J., Liu J., Chen Y. (2026). Polysaccharide-based injectable hydrogels for targeted drug delivery: Advances and challenges. Int. J. Pharm..

[22] Liang Z., Chen A., Lin H., Dong C., Huang Q., Li L., Chen J., Zhang W., Huang X., Wang J. (2026). A facile polysaccharide hydrogel activates PPARγ via the Gut-Kidney axis to ameliorate chronic kidney disease. J. Nanobiotechnol..

[23] Sang Z., Zhang W., Zhou Z., Fu H., Tan Y., Sui K., Xia Y. (2017). Functionalized alginate with liquid-like behaviors and its application in wet-spinning. Carbohydr. Polym..

[24] Yang J.-J., Zhang X., Dai J.-F., Ma Y.-G., Jiang J.-G. (2023). Effect of fermentation modification on the physicochemical characteristics and anti-aging related activities of Polygonatum kingianum polysaccharides. Int. J. Biol. Macromol..

[25] Teixeira F., Rut A., Costa P.C., Rodrigues F., Estevinho B.N. (2025). Design of Polymeric Delivery Systems for Lycium barbarum Phytochemicals: A Spray Drying Approach for Nutraceuticals. Foods.

[26] Li Y., Qiu Y., Hou H., Zhang G., Hao H., Bi J. (2023). The Preparation and Properties of Amino-Carboxymethyl Chitosan-Based Antibacterial Hydrogel Loaded with ε-Polylysine. Foods.

[27] Lombardo G., Dorm B.C., Salvay A.G., Franzi L., Gaffney M.L., Peredo Camio J.B., Trovatti E., Rossi E., Errea M.I. (2024). Novel chitosan-based hydrogels as promising wound dressing materials with advanced properties. Int. J. Biol. Macromol..

[28] Xie Y., Li G., Wu J., Zhu J., Cai X., Zhao P., Zhang D., Zhong Y. (2025). Injectable self-healing alginate/PEG hydrogels cross-linked via thiol-Michael addition bonds for hemostasis and wound healing. Carbohydr. Polym..

[29] Tang D.-X., Zhou S.-Z., Wang Z.-J., Zhang Y.-L., Liu K., Yang J.-Y., Yang X.-J., Li Y.-S., Zhang X. (2025). Efficiently radiation-induced degradation and extraction of Polygonatum sibiricum polysaccharides and its excellent ROS scavenging properties. Chem. Eng. J..

[30] Wang L., Dong C., Wang Q., Teng C., Gao W., He Y., Liang J., Xia Y., Kuang H., Sun Y. (2025). Polysaccharides from Actinidia Lindl.: Extraction, bioactivities, and emerging applications in food and pharmaceutical industries. Trends Food Sci. Technol..

[31] Chu Q., Xie S., Wei H., Tian X., Tang Z., Li D., Liu Y. (2024). Enzyme-assisted ultrasonic extraction of total flavonoids and extraction polysaccharides in residue from *Abelmoschus manihot* (L). Ultrason. Sonochem..

[32] Bono F., Puiggalí-Jou A., Lucherini L., Cocchi G., Zenobi-Wong M., Amstad E. (2026). Enzyme-induced mineralization of calcium carbonate in 3D printable granular hydrogels. Adv. Compos. Hybrid Mater..

[33] Lan X., Luo M., Li M., Mu L., Li G., Chen G., He Z., Xiao J. (2024). Swim bladder-derived biomaterials: Structures, compositions, properties, modifications, and biomedical applications. J. Nanobiotechnol..

[34] Torresan V., Gandin A., Contessotto P., Zanconato F., Brusatin G. (2025). Injectable hyaluronic acid-based hydrogel niches to create localized and time-controlled therapy delivery. Mater. Today Bio.

[35] Li Y., Li T., Feng J., Liu B., Wang Z., He J., Chen Z., Tao R., Wang H., Fan K. (2025). Acid-responsive contractile hyaluronic acid-based hydrogel loaded with ginsenoside Rg1 for hemostasis and promotion of gastric wound healing. Biomaterials.

[36] Paone L.S., Jin Y., Bouyer J., Fischer I., Galie P.A. (2026). Development of a multifunctional, injectable biomaterial using hyaluronan as a bioactive nanocarrier. Biomaterials.

[37] Choi J., Hwang D.S., Lim C., Lee D.W. (2024). Interaction mechanism between low molecular weight chitosan nanofilm and functionalized surfaces in aqueous solutions. Carbohydr. Polym..

[38] Qiao C., Ma X., Wang X., Liu L. (2021). Structure and properties of chitosan films: Effect of the type of solvent acid. LWT.

[39] Mi Y., Zhang J., Chen Y., Sun X., Tan W., Li Q., Guo Z. (2020). New synthetic chitosan derivatives bearing benzenoid/heterocyclic moieties with enhanced antioxidant and antifungal activities. Carbohydr. Polym..

[40] Firuzeh M., Labbaf S., Enayati M.H., Dinari M., Mirhaj M. (2025). Enhanced wound healing with a bilayered multifunctional quaternized chitosan-dextran-curcumin construct. Carbohydr. Polym..

[41] Trombino S., Serini S., Cassano R., Calviello G. (2019). Xanthan gum-based materials for omega-3 PUFA delivery: Preparation, characterization and antineoplastic activity evaluation. Carbohydr. Polym..

[42] Yan F., Xu Y., Long X., Chen Q. (2025). Ultrasound-enzyme-assisted aqueous two-phase extraction, structural characterization and bioactivities of Coptis chinensis polysaccharides. LWT.

[43] Li D., Wan Ibadullah W.Z., Shukri R., Duan Q., Gu Y., Mustapha N.A. (2025). The Effects of Different Extraction Methods on the Yield, Microstructure, and Antioxidant Activity of Polysaccharides from Rhodomyrtus tomentosa Berry. Food Bioprocess Technol..

[44] Amorim S., Reis C.A., Reis R.L., Pires R.A. (2021). Extracellular Matrix Mimics Using Hyaluronan-Based Biomaterials. Trends Biotechnol..

[45] Graça M.F.P., Miguel S.P., Cabral C.S.D., Correia I.J. (2020). Hyaluronic acid—Based wound dressings: A review. Carbohydr. Polym..

[46] Chen S.-K., Liu J.-J., Wang X., Luo H., He W.-W., Song X.-X., Nie S.-P., Yin J.-Y. (2024). Hericium erinaceus β-glucan/tannic acid hydrogels based on physical cross-linking and hydrogen bonding strategies for accelerating wound healing. Int. J. Biol. Macromol..

[47] Hu X., Wang Y., Zhang L., Xu M. (2020). Formation of self-assembled polyelectrolyte complex hydrogel derived from salecan and chitosan for sustained release of Vitamin C. Carbohydr. Polym..

[48] Wang P., Zhang Q., Wang S., Wang D., Yip R.C.S., Xie W., Chen H. (2025). Injectable Salecan/hyaluronic acid-based hydrogels with antibacterial, rapid self-healing, pH-responsive and controllable drug release capability for infected wound repair. Carbohydr. Polym..

[49] Mahmood H., Khan I.U., Asif M., Khan R.U., Asghar S., Khalid I., Khalid S.H., Irfan M., Rehman F., Shahzad Y. (2021). In vitro and in vivo evaluation of gellan gum hydrogel films: Assessing the co impact of therapeutic oils and ofloxacin on wound healing. Int. J. Biol. Macromol..

[50] Prateeksha, Sharma V.K., Liu X., Oyarzún D.A., Abdel-Azeem A.M., Atanasov A.G., Hesham A.E.-L., Barik S.K., Gupta V.K., Singh B.N. (2022). Microbial polysaccharides: An emerging family of natural biomaterials for cancer therapy and diagnostics. Semin. Cancer Biol..

[51] Qing X., Liu Z., Katsaounis A., Bouropoulos N., Taurino I., Fardim P. (2024). Poly(vinyl alcohol)/Pullulan/NaCl Conductive Hydrogels with High Strength and Sensitivity for Wearable Strain Sensors. ACS Appl. Polym. Mater..

[52] Zhang Z.-F., Ling M.-Y., Ge X.-R., Chen J.-Y., Zhang B., Yao X.-H., Song P., Zhao W.-G., Zhang D.-Y., Wang J. (2025). Dual-stage reverse-dialysis-engineered cartilage-biomimetic hydrogels: Highly resilient anti-swelling double-layer architectures. Chem. Eng. J..

[53] Zhao J., Chen R., Cheng D., Yang X., Zhang H., Zheng J., Hu R. (2025). Extremely Ultrahigh Stretchable Starch-Based Hydrogels with Continuous Hydrogen Bonding. Adv. Funct. Mater..

[54] Wang Y., Zhang Y., Zhong H., Guo M., Chen X., Lu Y. (2025). Construction of a non-toxic interpenetrating network hydrogel drug carrier supported by carbon microspheres and nanocellulose. Carbohydr. Polym..

[55] Zhu Y., Chen F., Wu B., Tong Y., Ning K., Zhen Z., Chen J. (2026). Supramolecular self-assembly of an herbal fructan and chitosan hydrogel: Analysis of the fructan, gel structures and the molecular interaction mechanisms. Carbohydr. Polym..

[56] Kang D.-H., Wang S., Goh M., Park J., Na H., Lee W.-J., Kim Y., Rahman M.S., Tae G., Yoon M.-H. (2024). Synthesis of Superabsorbent Hydrogels with Predefined Geometries and Controlled Swelling Properties for Versatile 3D Cell Culture Scaffolds. ACS Appl. Mater. Interfaces.

[57] Dave P.N., Bamaniya S., Singh P. (2026). Synthesis, characterization, and reinforcement effect of graphene oxide on rheological behaviour of gum acacia-grafted-poly(N,N-dimethylacrylamide) hydrogels. Int. J. Biol. Macromol..

[58] Syed Ahamed Hussain I., Jaisankar V. (2017). An eco-friendly synthesis, characterization and antibacterial applications of novel almond gum—poly(acrylamide) based hydrogel silver nanocomposite. Polym. Test..

[59] Leite A.C., Vicente A.A., Pereira R.N., Mendes C., Rodrigues R.M. (2026). Production and characterization of potato protein-based cryogels produced by different gelation mechanisms. Food Hydrocoll..

[60] Hu X., Zeng F., Yang Y., Hao B., Sun L., Wang T., Liu J., Xu H. (2025). Injectable enzyme-crosslinking antioxidant hydrogel for stem cells protection and application in skeletal muscle regeneration. Chem. Eng. J..

[61] Yan Y., Lv S., Qiang Y., She J., Cao X., He T., Liu L. (2026). Fabrication of CA–K+/P(AM–MBA)/AgNPs–K+ hydrogel with ion-electron synergistic conduction and ion-covalent dual-network enhancement and its flexible sensing performance. Carbohydr. Polym..

[62] Torabi Rahvar P., Abdekhodaie M.J., Jooybar E., Gantenbein B. (2024). An enzymatically crosslinked collagen type II/hyaluronic acid hybrid hydrogel: A biomimetic cell delivery system for cartilage tissue engineering. Int. J. Biol. Macromol..

[63] Zhang W., Liu S., Wang L., Li B., Xie M., Deng Y., Zhang J., Zeng H., Qiu L., Huang L. (2024). Triple-crosslinked double-network alginate/dextran/dendrimer hydrogel with tunable mechanical and adhesive properties: A potential candidate for sutureless keratoplasty. Carbohydr. Polym..

[64] Tavakoli M., Zare F. (2026). Electron beam crosslinked PVA/PAM/GO/SA hydrogel for high-efficiency solar desalination: Synergistic design and performance optimization. Sol. Energy Mater. Sol. Cells.

[65] Zhu S., Li Y., He Z., Ji L., Zhang W., Tong Y., Luo J., Yu D., Zhang Q., Bi Q. (2022). Advanced injectable hydrogels for cartilage tissue engineering. Front. Bioeng. Biotechnol..

[66] Liu Z., Liu C., Sun X., Zhang S., Yuan Y., Wang D., Xu Y. (2020). Fabrication and characterization of cold-gelation whey protein-chitosan complex hydrogels for the controlled release of curcumin. Food Hydrocoll..

[67] Xuan H., Liu Z., Lu K., Chen Y., Gu H., Li B., Sun J., Jin Y., Yang Y., Yuan H. (2026). Immunomodulatory hydrogel reprograms IL-17/NF-κB signaling to drive regeneration in diabetic wounds. Mater. Today Bio.

[68] Wang T., Wen M., Li N., Zhang L., Xue Y., Shang L. (2024). Marine-Derived Nanozyme-Crosslinked Self-Adaptive Hydrogels for Programmed Regulating the Regeneration Process. Adv. Funct. Mater..

[69] Hendriks J., Zoetebier B., Larrea C.S., Le N.X.T., Saris D.B.F., Karperien M. (2024). Gelatin-tyramine addition and low hydrogel density improves cell attachment, migration, and metabolic activity in vitro and tissue response in vivo in enzymatically crosslinkable dextran-hyaluronic acid hydrogels. Int. J. Biol. Macromol..

[70] Wu C., Yang F., Zhong H., Hong J., Lin H., Zong M., Ren H., Zhao S., Chen Y., Shi Z. (2024). Obesity-enriched gut microbe degrades myo-inositol and promotes lipid absorption. Cell Host Microbe.

[71] Lin Y., Lau H.C.-H., Liu C., Ding X., Sun Y., Rong J., Zhang X., Wang L., Yuan K., Miao Y. (2025). Multi-cohort analysis reveals colorectal cancer tumor location-associated fecal microbiota and their clinical impact. Cell Host Microbe.

[72] Yang K., Yang J., Chen R., Dong Q., Yang H., Gu S., Zhou Y. (2024). Antibacterial hyaluronic acid hydrogels with enhanced self-healing properties via multiple dynamic bond crosslinking. Int. J. Biol. Macromol..

[73] Du N., Chang Z., Li W., Li P., Lei F., Yao X., Li J., Wang M., Ma N., Jiang J. (2026). Alcohol-induced and ion-mediated strategy for flexible polysaccharide-based hydrogels with superior evaporative cooling performance. Carbohydr. Polym..

[74] Gan D., Jiang Y., Hu Y., Wang X., Wang Q., Wang K., Xie C., Han L., Lu X. (2022). Mussel-inspired extracellular matrix-mimicking hydrogel scaffold with high cell affinity and immunomodulation ability for growth factor-free cartilage regeneration. J. Orthop. Transl..

[75] Liu J., Li J., Yu F., Zhao Y.-x., Mo X.-m., Pan J.-f. (2020). In situ forming hydrogel of natural polysaccharides through Schiff base reaction for soft tissue adhesive and hemostasis. Int. J. Biol. Macromol..

[76] Xing D., Du Y., Dai K., Lang S., Bai Y., Liu G. (2024). Polysaccharide-Based Injectable Hydrogel Loaded with Quercetin Promotes Scarless Healing of Burn Wounds by Reducing Inflammation. Biomacromolecules.

[77] Xu D., Qiao T., Zhou Y.-M., Wu X.-Y., Cui Y.-L. (2025). A brain-targeted and ROS-responsive natural polysaccharide nanogel for enhancing antidepressant therapy. Chem. Eng. J..

[78] Jeong J.-p., Kim K., Oh E., Park S., Jung S. (2025). Self-Healing Hydrogels with Intrinsic Antioxidant and Antibacterial Properties Based on Oxidized Hydroxybutanoyl Glycan and Quaternized Carboxymethyl Chitosan for pH-Responsive Drug Delivery. Gels.

[79] Liu W., Wang X., Fan X., Zhou D., Hu W., Liu X., Zhu J. (2023). Glucose-Triggered Release of trans-N-p-Coumaroyltyramine from Zeolitic Imidazolate Framework-8 (ZIF8) Modified with Dioscorea opposita Thunb Polysaccharide. Langmuir.

[80] Deng K., Huang Q., Yan X., Dai Y., Zhao J., Xiong X., Wang H., Chen X., Chen P., Liu L. (2024). Facile fabrication of a novel, photodetachable salecan-based hydrogel dressing with self-healing, injectable, and antibacterial properties based on metal coordination. Int. J. Biol. Macromol..

[81] Chen S., Hou Z., Xiao M., Wu P., Yang Y., Han S., Xia J., Hu J., Zhang K., Yang L. (2025). Quaternized chitosan-based photothermal antibacterial hydrogel with pro-vascularization and on-demand degradation properties for enhanced infected wound healing. Carbohydr. Polym..

[82] Luo J., Wu Z., Zhao X., Qiao L., Huang Y., Chen J., Shan Y., Deng Z., Liang Z., Han Y. (2025). Network-Remodeling, Electroactive and Antibacterial Cryogel with Tissue Sealing and Pro-Coagulant Activity for Hemostasis and Skin Wound Healing. Adv. Funct. Mater..

[83] Han X., Lu T., Zhang Z., Wang H., Lu S. (2023). Tremella polysaccharide-based conductive hydrogel with anti-freezing and self-healing ability for motion monitoring and intelligent interaction. Int. J. Biol. Macromol..

[84] Yan X., Chen Y., Dan N., Dan W. (2022). A novel thermosensitive growth-promoting collagen fibers composite hemostatic gel. J. Mater. Chem. B.

[85] Wang S., Fu Q., Cen W., Su Z., Jin W., Yu Z., Xu S. (2025). A multifunctional hydrogel based on Sanghuang polysaccharides and MXene for infected wound healing. Chem. Eng. J..

[86] Shakibi R., Khayamian M.A., Abadijoo H., Dashtianeh M., Kolahdouz M., Daemi H., Abdolmaleki P. (2024). Enhancing cell activities through integration of polyanionic alginate or hyaluronic acid derivatives with triboelectric nanogenerators. Carbohydr. Polym..

[87] Wu J., Xue W., Yun Z., Liu Q., Sun X. (2024). Biomedical applications of stimuli-responsive “smart” interpenetrating polymer network hydrogels. Mater. Today Bio.

[88] Wu D., Lin Q., Wang Z., Huang H., Song X., Gao Y., Yang X., Wen K., Sun X. (2024). Mechanism of Xue-Jie-San treating Crohn’s disease complicated by atherosclerosis: Network pharmacology, molecular docking and experimental validation. Phytomedicine.

[89] Pedro S.N., Valente B.F.A., Vilela C., Oliveira H., Almeida A., Freire M.G., Silvestre A.J.D., Freire C.S.R. (2023). Switchable adhesive films of pullulan loaded with a deep eutectic solvent-curcumin formulation for the photodynamic treatment of drug-resistant skin infections. Mater. Today Bio.

[90] Wang W., Liu Q., Lv A., Lin Y., Chen J., Ding N., Zhang C., Zhou S., Yuan B., Zhao W. (2026). Bioactive Conductive Ti3C2Tx-Ce Hydrogel Facilitates Spinal Cord Injury Repair Through ROS Scavenging and Mitochondrial Regulation. Adv. Funct. Mater..

[91] Fang Y., Zhang L., Chen Y., Wu S., Weng Y., Liu H. (2023). Polysaccharides based rapid self-crosslinking and wet tissue adhesive hemostatic powders for effective hemostasis. Carbohydr. Polym..

[92] Xu J., Cui Y., Sun X., Chen Z., Liu M., Wang X., Li P., Fan Y. (2025). Continuous magnetic-gradient hydrogel with augmented mechanical span and reverse-directional polysaccharides distribution for integrated repair of osteochondral defects. Compos. Part B Eng..

[93] Luo L., Zhang S., Gong J., Zhang J., Xie P., Yin J., Zhang M., Zhang C., Chen H., Liu Y. (2025). 3-D Sustained-Release Culture Carrier Alleviates Rat Intervertebral Disc Degeneration by Targeting STING in Transplanted Skeletal Stem Cells. Adv. Sci..

[94] Archana M., Rubini D., Dharshini K.P., Hari B.N.V., Jayasankari S., Ramyadevi D., Gonciarz W., Domańska A., Brzeziński M., Nithyanand P. (2023). Development of an anti-infective urinary catheter composed of polyvinyl alcohol/sodium alginate/methylcellulose/polyethylene glycol by using a pressure-assisted 3D-printing technique. Int. J. Biol. Macromol..

[95] Zhong J., Tang Y., Hu X., Deng X., Liu M., Cao Y., Peng L., Liu W., Liu X., Deng L. (2026). Dextran-based microneedle patch Co-delivering safflower polysaccharide and ROS-responsive tetramethylpyrazine micelles for diabetic wound repair. Mater. Today Bio.

[96] Fu W., Sun S., Cheng Y., Ma J., Hu Y., Yang Z., Yao H., Zhang Z. (2024). Opportunities and challenges of nanomaterials in wound healing: Advances, mechanisms, and perspectives. Chem. Eng. J..

[97] Park M., Tripathi G., Fahad M.A.A., Park S.-s., Gwon J.-G., Cho H., Kim D.H., Lee B.-T. (2026). MSCs-loaded injectable multifunctional dSECM hydrogels enhance wound healing through the activation of Wnt signaling pathways. Adv. Compos. Hybrid Mater..

[98] Das I.J., Bal T. (2024). pH factors in chronic wound and pH-responsive polysaccharide-based hydrogel dressings. Int. J. Biol. Macromol..

[99] Haidari H., Vasilev K., Cowin A.J., Kopecki Z. (2022). Bacteria-Activated Dual pH- and Temperature-Responsive Hydrogel for Targeted Elimination of Infection and Improved Wound Healing. ACS Appl. Mater. Interfaces.

[100] Deng Z., Guo Y., Wang X., Song J., Yang G., Shen L., Wang Y., Zhao X., Guo B., Wang W. (2025). Multiple crosslinked, self-healing, and shape-adaptable hydrogel laden with pain-relieving chitosan@borneol nanoparticles for infected burn wound healing. Theranostics.

[101] Lin C., Yang M., Zhang F., Liu Y., Leng J. (2026). Stimuli-responsive smart materials for biomedical applications. Mater. Sci. Eng. R Rep..

[102] Ma Z., Yang X., Ma J., Lv J., He J., Jia D., Qu Y., Chen G., Yan H., Zeng R. (2021). Development of the mussel-inspired pH-responsive hydrogel based on Bletilla striata polysaccharide with enhanced adhesiveness and antioxidant properties. Colloids Surf. B Biointerfaces.

[103] Chen Y., Lin Y., Liu Z., Zhang Y., Hu Z., Ai M., Shen Y., Lian D., Wang C., Liu X. (2025). ROS-responsive adaptive injectable hydrogel promoting inflammatory mastoid bone repair through efficient sterilization and regulating oxidative stress and macrophage phenotype. Mater. Today Bio.

[104] Ding S., Yu X., Wang Q., Luo P., Li H., Li Z., Wang R., Liu H., He Y., Nong J. (2025). Multifunctional hydrogel sensors with dynamic covalent networks for machine learning-assisted Parkinson’s disease diagnosis and encrypted human-computer interaction. Mater. Today Bio.

[105] Ding A., Tang F., Ayyagari S., Alsberg E. (2026). Reprogrammable 4D tissue engineering hydrogel scaffold via reversible ion printing. Bioact. Mater..

